# Probabilistic model for individual assessment of central hyperexcitability using the nociceptive withdrawal reflex: a biomarker for chronic low back and neck pain

**DOI:** 10.1186/1471-2202-14-110

**Published:** 2013-10-03

**Authors:** José A Biurrun Manresa, Giang P Nguyen, Michele Curatolo, Thomas B Moeslund, Ole K Andersen

**Affiliations:** 1 Center for Sensory-Motor Interaction, Department of Health Science and Technology, Aalborg University, Fredrik Bajers Vej 7, Aalborg, Øst 9220, Denmark; 2 Department of Anesthesiology and Pain Medicine, University of Washington, 1959 NE Pacific Street, BB-1469, Box 356540, Seattle, WA 98195-6540, USA; 3 Computer Vision and Media Technology Laboratory, Department of Architecture, Design and Media Technology, Aalborg University, Niels Jernes Vej 14, Aalborg, Øst 9220, Denmark

**Keywords:** Nociceptive withdrawal reflex, Chronic pain, Biomarker, Machine learning, Pattern recognition, EMG classification

## Abstract

**Background:**

The nociceptive withdrawal reflex (NWR) has been proven to be a valuable tool in the objective assessment of central hyperexcitability in the nociceptive system at spinal level that is present in some chronic pain disorders, particularly chronic low back and neck pain. However, most of the studies on objective assessment of central hyperexcitability focus on population differences between patients and healthy individuals and do not provide tools for individual assessment. In this study, a prediction model was developed to objectively assess central hyperexcitability in individuals. The method is based on statistical properties of the EMG signals associated with the nociceptive withdrawal reflex. The model also supports individualized assessment of patients, including an estimation of the confidence of the predicted result.

**Results:**

up to 80% classification rates were achieved when differentiating between healthy volunteers and chronic low back and neck pain patients. EMG signals recorded after stimulation of the anterolateral and heel regions and of the sole of the foot presented the best prediction rates.

**Conclusions:**

A prediction model was proposed and successfully tested as a new approach for objective assessment of central hyperexcitability in the nociceptive system, based on statistical properties of EMG signals recorded after eliciting the NWR. Therefore, the present statistical prediction model constitutes a first step towards potential applications in clinical practice.

## Background

Chronic pain states are associated with changes in the nociceptive system that may lead to hypersensitivity, i.e., pain after innocuous stimulation or exaggerated pain after low-intensity nociceptive stimulation [1]. Patients with chronic pain syndromes, such as whiplash, fibromyalgia, osteoarthritis, basal ganglia disorders, migraine and tension-type headache, endometriosis or chronic low back pain may display such pain hypersensitivity after stimulation of healthy tissues, most likely resulting from increased excitability in the central processing of sensory input [2,3].

The assessment of these conditions may be hampered by the subjective and unreliable nature of self-report based instruments. The establishment of objective, affordable and reliable measures of pain hypersensitivity would advance the understanding of neural mechanisms behind chronic pain, provide a basis for improved clinical management of pain, and establish much needed objective measures of treatment success or failure [4].

In this regard, the nociceptive withdrawal reflex (NWR) is a potential biomarker that has already been proven useful in the assessment of physiological, chemical and pharmacological modulation of nociceptive transmission/processing [5-9]. Moreover, it has been shown that the NWR is a valuable tool in the objective assessment of central hyperexcitability in the spinal nociceptive system that is present in many chronic pain disorders [7,9]. However, most of the studies on objective assessment of central hyperexcitability focus on population differences between patients and healthy individuals and do not provide tools for individual assessment [7,8].

The aim of this study was to develop a method to provide objective and individual assessment of central hyperexcitability using a biomarker derived from the NWR. In order to accomplish this, data from chronic low back and neck pain patients and healthy subjects was used to construct and test a prediction model based on statistical properties of the NWR signals. The methods and results of this model are presented and the relevance of such biomarker is discussed in the context of individual assessment of central hyperexcitability in clinical settings.

## Methods

### Participants

Data from 280 subjects was collected and divided in two groups. One group contained data from 140 patients (70 males and 70 females) with chronic low back pain or chronic neck pain. Inclusion criteria for chronic pain patients were: daily pain of at least 6 months duration and pain at the time of testing with an intensity of at least 3 using a 10-cm visual analogue scale (VAS), whereby 0 = no pain and 10 = worst pain imaginable. Exclusion criteria for chronic pain patients were: radicular pain (as defined by leg pain associated with a magnetic resonance imaging finding of herniated disc or foraminal stenosis with contact to a nerve root); peripheral or central neurological disorders, diabetes mellitus, insufficient knowledge of the German language, pregnancy (as ruled out by pregnancy test), breast feeding, intake of oral contraceptives or hormones, intake of opioids and antidepressants during the previous 2 weeks, and intake of other analgesics during the 48 hours before testing. The second group contained data from 140 healthy subjects (70 males and 70 females). Exclusion criteria for healthy subjects were the same as for the patient group, plus any pain at the time of testing or history of chronic pain syndrome of any nature. Both groups were evenly distributed with respect to gender and age. The age of the subjects ranged from 20 to 80 years with a mean age of 50 years. Both groups were tested using the same experimental setup for the recording of the NWR. All subjects were recruited at the University Hospital of Bern, Inselspital. The dataset was originally collected in order to establish reference values for the NWR and reflex receptive fields (RRF, the area from which the NWR can be elicited) in healthy subjects, and to determine if there are population differences compared to chronic pain patients (for further details, please refer to [9-12]). Written informed consent was obtained prior to participation and the Declaration of Helsinki was respected. The study was approved by the local ethics committee of the Canton of Bern (KEK 147/04).

### Setup

#### ***Electrical stimulation***

During testing, participants were lying in a bed, in a quiet room. A leg rest was placed under the knees to obtain a 30º semiflexion during electrophysiological testing. Ten surface stimulation electrodes (15 x 15 mm, type Neuroline 700, Ambu A/S, Denmark) were non-uniformly mounted on the sole of the foot and a common anode (5 x 9 cm) was placed on the dorsum of the foot, which are subsequently denoted as sites 1,2,…,10. Each stimulus consisted of a constant-current pulse train of 5 individual 1-ms pulses delivered at 200 Hz (Noxitest IES 230 stimulator, Aalborg, Denmark). This stimulus is perceived by the subjects as a single pricking/pinching stimulus, and potentially evokes a single ankle dorsiflexion/plantarflexion withdrawal reaction (depending on the stimulation site). A computer-controlled stimulator delivered a stimulus to one electrode at a time in a randomized order, with a random inter-stimulus interval ranging from 10 to 15 s. For each electrode site, the lowest stimulus intensity that evoked pain (i.e., the pain threshold) was assessed using a staircase procedure, and a stimulation intensity of 1.5 times the pain threshold was selected for eliciting the NWR [13], in order to ensure that a NWR response was evoked by most of the stimulations. Each electrode site was stimulated 4 times.

#### ***Signal recording***

**Figure 1:**
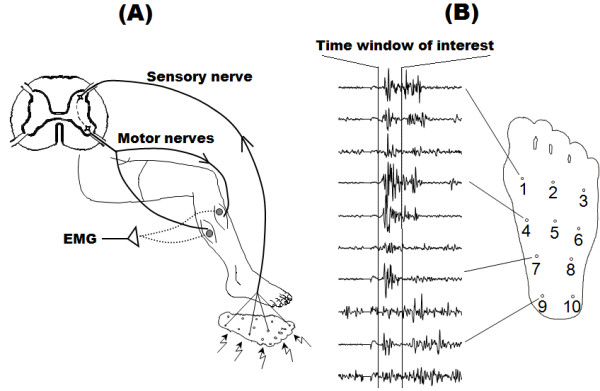
**Methodology for NWR stimulation and recording in humans**. **(A)** Reflex responses evoked by distributed electrical stimulation on the sole of the foot were recorded by surface EMG at selected muscles. **(B)** The reflex size was quantified in the time windows of interest (usually 60–180 ms after stimulation).

Activity in the tibialis anterior muscle was measured using surface electromyography (EMG), as can be seen in Figure 1.A. Initially the skin was lightly abraded, and then two surface electrodes (30 x 22 mm, type Neuroline 720, Ambu A/S, Denmark) were placed along the muscle fiber direction over the muscle with an inter-electrode distance of 20 mm. The signal was amplified (up to 20000 times) and filtered (5–500 Hz, 2nd order) by custom-made EMG amplifiers (Aalborg University, Denmark). Afterwards, it was sampled (2000 Hz) and stored (1000 ms window including 200 ms of pre-stimulation activity, commonly used to verify that there is no muscle activity before the stimulation), using a software specifically developed for NWR acquisition and analysis [14]. The NWR was quantified within the 60–180 ms post-stimulation interval (Figure 1.B).

### Prediction model

#### ***Data preparation***

Data was divided into three disjoint subsets: training set (**TR**), validation set (**V**) and test set (**TE**) [15-17]. The training set **TR** contains the data from which the model was derived. The validation set **V** was used to adjust and find the optimal model. Finally, the test set **TE** was used to assess the performance of the optimal model. Data was split as 70%-15%-15% of the whole dataset for **TR** (200 subjects), **V** (40 subjects) and **TE** (40 subjects) sets, respectively.

Two restrictions were applied when assigning subjects into each set. First, the number of patients and healthy subjects should be balanced. Second, subjects within each group (patient or healthy) should be distributed evenly with respect to gender and age. Assuming a data vector of *N* samples $X={\left\{X_{i}\right\}}_{i=\bar{1,N}}$ from which *n* samples need to be selected following the aforementioned restrictions; two age classes: *A*_1_ = {*X*_*i*_ < 50*y*} and *A*_2_ = {*X*_*i*_ ≥ 50*y*}, and two genders *G*_1_ = {*X*_*i*_ is male} and *G*_1_ = {*X*_*i*_ is female} were included. The set **S** was derived as:

$$S=S_{1}\cup S_{2}\cup S_{3}\cup S_{4}$$

where

$$\begin{array}{c} S_{1}=A_{1}\cap G_{1}\;S_{2}=A_{1}\cap G_{2}\\ S_{3}=A_{2}\cap G_{1}\;S_{4}=A_{2}\cap G_{2} \end{array}$$

and ‖Si‖i=1..4=n4. Following these restrictions, the data was finally divided as shown in Table 1.

**Table 1:** Data preparation

	**Healthy**				**Patient**			
	**A**_**1**_	**A**_**2**_	**G**_**1**_	**G**_**2**_	**A**_**1**_	**A**_**2**_	**G**_**1**_	**G**_**2**_
**TR**	50	50	50	50	50	50	50	50
**V**	10	10	10	10	10	10	10	10
**TE**	10	10	10	10	10	10	10	10

All 140 patients and 140 healthy subjects were divided into a training set (**TR**), a validation set (**V**) and a test set (**TE**). Each set satisfied two criteria: balance between patients and healthy subjects, and even distribution on gender and age.

#### ***Feature selection***

Several features were derived from the EMG recordings of the NWR in order to perform an initial exploratory analysis of their discriminative capacity. Preliminary tests showed promising results using the EMG amplitudes of the NWR, in line with previous investigations [18-21]. Specifically, the exploratory analysis showed some differences between the probability distributions of EMG amplitudes between patients and healthy subjects, so it was hypothesized that those differences could be used for classification purposes.

**Figure 2:**
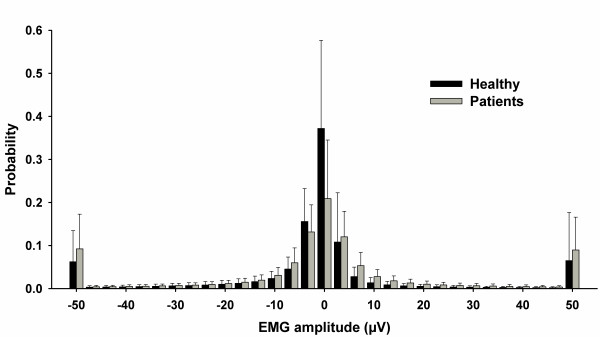
**Average histogram of EMG signals from all patients and healthy subjects. Error bars represent standard deviation**.

Since the stimulation was repeated four times at each site, each sample *X*_*i*_ ∈ **X** had four signals $F_{\mathrm{ij}},j=\overset{¯}{1,4}$. For each signal, a probability distribution histogram was constructed to be used as classification feature. To compute a probability distribution histogram, it was required to determine the number of sub-ranges or bins that should be used. To this end, "EMG amplitudes of all signals {*F*_*ij*_} from all training samples *X*_*i*_ were taken into account," where *X*_*i*_ ∈ **TR**. The EMG amplitude range was defined as *Rg* = [*min* (*F*_*ij*_), *max* (*F*_*ij*_)]. Since the EMG signal values have rather large range *Rg*, the selected number of bins should be reasonably high. Therefore, different numbers of bins, namely 100, 150, 200 and 300 were tested. The experiments showed that in all the cases, there were very few extreme EMG amplitude values, in the order of ~1/1000, which means that the probability distribution of EMG amplitudes in the area of the two ends of *Rg* is ~0. The data was mainly distributed within the range *rg* = [*μ-σ, μ + σ*], where *μ* and *σ* denote the mean and the standard deviation of {*F*_*ij*_}, respectively, so this new, restricted range was used. With the new range *rg* and selecting the model using a *leave*-*one*-*out* cross-validation procedure [22], only 30 bins were required to construct a probability distribution histogram, effectively reducing the number of features to be used as input to the classifier. Figure 2 shows the average histogram (an estimate of the probability distributions of EMG amplitudes) for patients and healthy volunteers.

#### ***Prediction scheme***

Given a set of training subjects {*X*_*i*_ ∈ **TR**}, which contain *N*^*h*^ and *N*^*p*^ training samples from healthy subjects and patients respectively, probability distributions *P*_*ij*_ were computed for each signal $F_{\mathrm{ij}},j=\overset{¯}{1,4}$ of a subject $X_{i},i=\overset{¯}{1,N^{h}+N^{p}}$. The subject *X*_*i*_ was represented by the probability *P*_*i*_, where:

$$P_{i}=\frac{\sum_{j=1}^{4}P_{\mathrm{ij}}}{4}$$

*X*_*i*_ was assigned with a label *L*_*i*_ = *h* if *X*_*i*_ was a healthy subject and *L*_*i*_ = *p* otherwise. When a query subject was sent to the prediction model to be classified as a patient or a healthy subject, the decision was made based on nearby training subjects with respect to the query subject. This approach is known as the *k*-nearest neighbour (kNN) [23]. kNN is an efficient algorithm in machine learning, showing comparable classification performance to more complex algorithms [16,24]. Briefly, when given a query subject, the kNN algorithm searches in the training set for the *k* subjects that are closest to the query, based on some predefined criterion to measure closeness (e.g. Euclidean distance). The query subject is then assigned to the group which has the majority among *k* subjects. Regarding the value of *k*, there is no specific approach for selecting an optimal value, as this strongly depends on the data structure [25]. Using *leave*-*one*-*out* cross-validation [22], a *k* value of 5 was finally selected.

The proposed prediction scheme was implemented as follows: given an unknown subject *X*_*q*_ (*X*_*q*_ ∈ **V** or *X*_*q*_ ∈ **TE**), a probability distribution *P*_*qj*_ was computed for each signal $F_{\mathrm{qj}},j=\overset{¯}{1,4}$. Then, each probability distribution *P*_*qj*_ was compared with all training probability distributions $P_{i},i=\overset{¯}{1,N^{h}+N^{p}}$. The Euclidean distance between these distributions was calculated as:

$$d_{\mathrm{ij}}=\sqrt{\overset{30}{\underset{x=1}{\sum }}{\left(P_{i}^{x}-P_{\mathrm{qj}}^{x}\right)}^{2}}$$

Where *k* training subjects with smallest distance were considered, and $x=\overset{¯}{1,30}$ represents the size of each classification feature (i.e. the 30 bins of each histogram). If the majority of these *k* training subjects had label *h* then the signal *F*_*qj*_ was labelled as healthy, and vice versa. Therefore, *X*_*q*_ had four predicted labels, one for each of the four signals *F*_*qj*_. The final prediction result for the unknown subject *X*_*q*_ was decided based on a voting mechanism, where the majority of votes was chosen to classify the subject as belonging to a patient or a healthy group. With four signals, there were cases where voting result was equal, i.e. 50:50 prediction results. From a clinical perspective, it is better to minimize the chance of missing any potential patients. Therefore, in case of equal voting, the unknown subject *X*_*q*_ was classified as a patient.

#### ***Individual assessment***

The prediction model also allows for individual assessment; as mentioned in the previous section, each signal *F*_*qj*_ of an unknown subject *X*_*q*_ returned a predicted label, according to the majority of *k* nearest neighbours. This label can be described as a probability value. The following formulas were used to compute the probability value for each signal *F*_*qj*_ to be labelled as patient or healthy, respectively:

$$\begin{array}{c} l_{\mathrm{qj}}^{p}=\frac{\left|L^{k}=p\right|}{k}\left(\%\right)\\ l_{\mathrm{qj}}^{h}=\frac{\left|L^{k}=h\right|}{k}\left(\%\right) \end{array}$$

Where *L*^*k*^ denotes the labels of *k* nearest neighbours at the *j*-th signal. For example, for the *j*-th signal, if all *k* nearest neighbours had the same label *p*, then that particular signal *F*_*qj*_ was labelled *p* with a probability of 100%. Another example: if ^3^/_5_ neighbours are labelled *p* and ^2^/_5_ neighbours are labelled *h*, then the signal *F*_*qj*_ was labelled *p* with probability of ~60%. In other words, the signal *F*_*qj*_ had ~60% probability of belonging to a chronic pain patient and ~40% probability of belonging to a healthy subject. With four signals for each subject, the individual assessment can be obtained by:

$$l_{q}^{p}=\frac{\sum l_{\mathrm{qj}}^{p}}{4}\left(\%\right)$$

**Figure 3:**
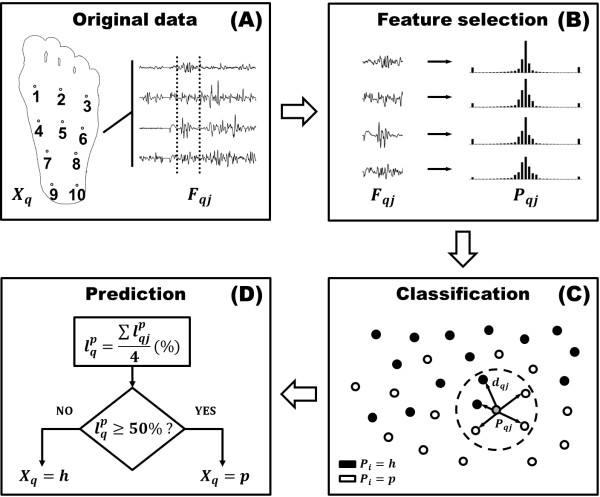
**Scheme of the proposed probabilistic prediction model. (A)** Given a query subject *X*_*q*_ ∈ **TE**, a set of EMG signals $F_{\mathrm{qj}},j=\overset{¯}{1,4}$ are obtained as a response to repeated electrical stimulation of ten sites on the sole of the foot. **(B)** A probability distribution histogram *P*_*qj*_ is constructed from each signal *F*_*qj*_ (or combination of signals from multiple sites) to be used as classification feature. **(C)** The signal *F*_*qj*_ is labelled *p* (for patient) or *h* (for healthy), depending on the distances *d*_*qj*_ to the closest neighbouring histograms *P*_*i*_, derived from the set of training subjects {*X*_*i*_ ∈ **TR**}. **(D)** The final prediction for the subject *X*_*q*_ is carried out based on the labels *l*_*qj*_ derived from the individual assessment of all four signals. Query subjects *X*_*q*_ ∈ **V** were used instead for all validation procedures (site combination and training set selection).

If $l_{q}^{p}$ is higher or equal to 50%, the query subject *X*_*q*_ is finally classified as patient, otherwise it is classified as healthy. Furthermore, the above value indicates how likely a subject is predicted as a patient: higher values result in higher confidence in the assessment. Figure 3 shows a general overview of the proposed prediction scheme.

### Model validation

The validation set **V** was used to find an optimal combination of different factors in order to achieve the highest prediction rate. As mentioned in the *Data preparation* section, 40 subjects (of which 20 are healthy subjects and 20 are patients) were included in **V**. Further experiments to validate the prediction model were conducted with different number of training subjects, since this number can influence the performance of the model [10]. Out of the 200 training subjects (100 patients and 100 healthy subjects), different subsets were extracted as a new training sets. *N*^*h*^ and *N*^*p*^ denote the number of selected training healthy subjects and patients, respectively. Since the training set had to follow the rules established in the *Data preparation* section, *N*^*h*^ and *N*^*p*^ were selected such that they were modulus 4:

$$\begin{array}{l} \left(N_{1}^{h},N_{1}^{p}\right)=\left(12,12\right)\;\left(N_{2}^{h},N_{2}^{p}\right)=\left(24,24\right)\\ \left(N_{3}^{h},N_{3}^{p}\right)=\left(32,32\right)\;\left(N_{4}^{h},N_{4}^{p}\right)=\left(48,48\right)\\ \left(N_{5}^{h},N_{5}^{p}\right)=\left(60,60\right)\;\left(N_{6}^{h},N_{6}^{p}\right)=\left(80,80\right)\\ \left(N_{7}^{h},N_{7}^{p}\right)=\left(100,100\right) \end{array}$$

When $N_{l}^{h}\;\mathrm{and}\;N_{l}^{p}$ < 100, more than one combination among training subjects to select a subset were available. For the model validation, 10 combinations were randomly chosen for each case. It should be noted that all combinations should obey the even distribution restrictions. Therefore, for each set of values $\left(N_{l}^{h},N_{l}^{p}\right),l=\bar{1,6}$, 10 training sets were collected, namely $TR_{t}^{l},t=\overset{¯}{1,10}$. The same validation set **V** was used in all cases to test the prediction model.

### Model evaluation

Different parameters might affect the performance of the prediction model, such as selection of training set, number of training subjects and stimulation sites (see next section). The validation set **V** was first used to tune these parameters. Once the model was optimized, the test set **TE** was used to evaluate the real performance of the model, without further parameter tuning. Since it is known in advance which subjects belong to the healthy group and which ones belong to the patient group, this knowledge was used to evaluate the prediction model. The evaluation of the model’s performance was based on prediction rates:

$$r=\frac{M^{+}}{M}\left(\%\right)$$

Where $M^{+}$ denotes the number of correct classified subjects among total number of subjects for validation or test, respectively, i.e. *M* = ‖**V**‖or *M* = **TE** depending on whether the validation set or the test set was used. Higher prediction rates indicate better performance of the model. To avoid cases where the prediction rate was high but most of the query subjects were classified as either patient or healthy, the previous equation was also applied to each group separately. In other words, it was extended as

$$r_{h}=\frac{M_{h}^{+}}{M_{h}}\left(\%\right)\;\mathrm{and}\;r_{p}=\frac{M_{p}^{+}}{M_{p}}\left(\%\right)$$

Where $M_{h}^{+}$ denotes the number of healthy subjects which were correctly classified and $M_{p}^{+}$ denotes the number of patients which were correctly classified. *M*_*h*_ and *M*_*p*_ are the total number of healthy subjects and patients for validation or test, respectively.

## Results

### Model validation

#### ***Stimulation site evaluation***

Several factors can influence the EMG signals recorded after stimulation of each site depending on the location of the electrode on the sole of the foot, such as skin thickness or nerve fiber density [24]. Eventually, sites with low prediction rates should be eliminated, meaning that fewer electrodes will be needed to place on a subject’s sole of the foot in a potential future application of the NWR as a biomarker for individual assessment of pain. Therefore, the first part of the model’s validation was carried out to compare the performance using each of the ten stimulation sites separately. Following the scheme (Figure 3), each subject from the validation set **V** was sent as input. kNN was applied with *k*=5. The average prediction rate *r* was reported. Given $\left(N_{l}^{h},N_{l}^{p}\right),l=\overset{¯}{1,6}$ training subjects, average prediction performance over 10 runs with $TR_{t}^{l},t=\overset{¯}{1,10}$ at each site was reported. As there was only one set of $\left(N_{7}^{h},N_{7}^{p}\right)$ training subjects, only one prediction result was obtained for this case (see Table 2).

**Table 2:** Average prediction rates at each site with different numbers of training subjects

	**(12,12)**	**(24,24)**	**(32,32)**	**(48,48)**	**(60,60)**	**(80,80)**	**(100,100)**	**Best performance**
**Site 9**	68%	68%	73%	73%	75%	77%	85%	85%
**Site 10**	64%	66%	69%	66%	67%	68%	80%	80%
**Site 3**	57%	62%	63%	69%	72%	75%	78%	78%
**Site 8**	57%	65%	61%	66%	67%	71%	63%	71%
**Site 1**	53%	59%	62%	61%	65%	69%	70%	70%
**Site 5**	51%	56%	58%	61%	60%	69%	65%	69%
**Site 2**	56%	63%	61%	62%	62%	64%	68%	68%
**Site 7**	59%	59%	58%	64%	63%	61%	63%	64%
**Site 4**	50%	53%	52%	59%	54%	58%	63%	63%
**Site 6**	51%	62%	55%	60%	59%	58%	53%	62%

Sites are sorted based on best performance in the last column.

The best performance at each site over different number of training subjects is displayed in Table 2. To decide which sites should be discarded, a threshold of 75% for the prediction rate was used. From this, 3 sites were selected whereas the remaining 7 were discarded (see Figure 1.B for an illustration of the locations of selected and eliminated sites). The final results on stimulation site evaluation suggested that signals recorded by stimulation of sites 3, 9 and 10 should be chosen for further evaluation.

#### ***Site combination***

Following with the model’s validation, the analysis was focused on the remaining 3 sites, namely sites {3, 9, 10}. Different combinations among those sites were also tested: {3, 9}, {3, 10}, {9, 10} and {3, 9, 10}. When more than one site was used, the voting mechanism was applied. Results are displayed in Table 3 for comparisons between single site and combinations of sites. Results showed that in general, the prediction rates were improved by combining sites compared to using a single site. Overall, the best performance (85% correct predictions) was reached by a combination of EMG signals recorded after stimulation of sites {9, 10}, {3, 9, 10} and {9}. The combination {9, 10} was finally chosen because it gave the best average prediction with different numbers of training subjects.

**Table 3:** Average prediction rates with different numbers of training subjects

	**(12,12)**	**(24,24)**	**(32,32)**	**(48,48)**	**(60,60)**	**(80,80)**	**(100,100)**	**Best performance**
**Sites** {**9**,**10**}	67%	70%	73%	73%	77%	77%	85%	85%
**Sites** {**3**,**9**,**10**}	64%	68%	67%	74%	77%	79%	85%	85%
**Site 9**	68%	68%	73%	73%	75%	77%	85%	85%
**Sites** {**3**,**10**}	64%	69%	69%	72%	78%	80%	80%	80%
**Site 10**	64%	66%	69%	66%	67%	68%	80%	80%
**Sites** {**3**,**9**}	67%	67%	70%	76%	77%	77%	78%	78%
**Site 3**	57%	62%	63%	69%	72%	75%	78%	78%

Prediction using four combinations of site were reported and compared to the classification performance of each of the three selected sites separately. Sites are sorted based on best performances displayed in the last column (when best performance is similar, sites are sorted based on average performance).

#### ***Training set selection***

**Figure 4:**
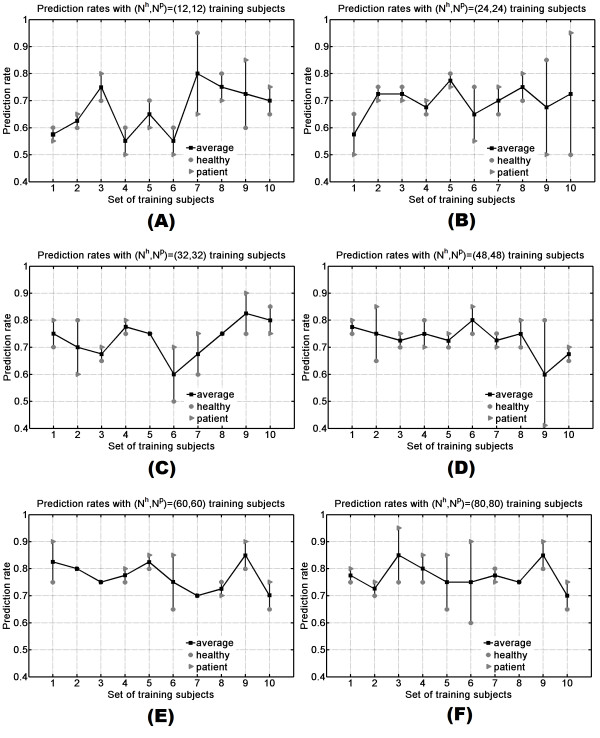
**Comparison of prediction rates for each set of training subjects.** Panels **A** to **F** show the average prediction performance of ten runs for each set $\left(N_{l}^{h},N_{l}^{p}\right),l=\overset{¯}{1,6}$, respectively.

In general, higher numbers of training samples do not always lead to better training model [15], since at a certain point the model will not improve further or even drop down in performance. To find an optimal number of training samples, an empirical approach is often used [24,26]. The performance of the prediction model was influenced by the number of training subjects, as shown in Tables 2 and 3. Moreover, with the same number of training subjects $\left(N_{l}^{h},N_{l}^{p}\right),l=\overset{¯}{1,6}$, different subset $TR_{t}^{l},t=\overset{¯}{1,10}$ also gave different prediction rates. With each set $\left(N_{l}^{h},N_{l}^{p}\right),l=\overset{¯}{1,6}$, three values were reported for a training set $TR_{t}^{l},t=\overset{¯}{1,10}$: *r*, *r*_*h*_ and *r*_*p*_, representing the average prediction rate, the prediction rate for healthy subjects and the prediction rate for patients, respectively. From previous validation results, a combination of EMG signals recorded after stimulation of sites 9 and 10 was used. Figure 4 shows the prediction result for each case, demonstrating that even with only 12 training subjects for each group, a well selected subset can reach up to 80% correct classified on average (Figure 4.A, *t* = 7). It was also observed that some of the training subsets did not perform very well, with prediction rates lower than 60%. In general, higher number of training subjects (48, 60 and 80, Figures 4.D, 4.E and 4.F, respectively) resulted in an improvement in the performance over lower number of training subjects (12, 24 and 32, Figures 4.A, 4.B and 4.C, respectively). With higher number of training subjects (48, 60 and 80), performances among different subsets were rather comparable. In case of $\left(N_{7}^{h},N_{7}^{p}\right)$ with *t* = 1 (only one training set), results were 75%, 95% and 85% for *r*_*p*_, *r*_*h*_ and *r*, respectively.

The case with highest prediction rate was selected for each set $\left(N_{l}^{h},N_{l}^{p}\right)$. As mentioned before, balance between *r*_*h*_ and *r*_*p*_ is also important. This means that the selected model should have a high average prediction rate *r*, and at the same time, the difference between *r*_*h*_ and *r*_*p*_ should be small. For instance, in Figure 4.B, at *t* = 10, the average prediction rate was *r* = 72% but difference between *r*_*h*_ = 50% and *r*_*p*_ = 95% was large, i.e., most of the subjects were predicted as patients. Therefore, this model should not be chosen. In Figure 4.F, average prediction rates at *t* = 3 and *t* = 9 were equal, but case *t* = 9 was selected because ‖*r*_*h*_ - *r*_*p*_‖ was smaller than in case *t* = 3. Comparison among best prediction rates with different numbers of training subjects is displayed in Table 4. The results show a tight competition between 3 sets $\left(N_{5}^{h},N_{5}^{p}\right)$, $\left(N_{6}^{h},N_{6}^{p}\right)$ and $\left(N_{7}^{h},N_{7}^{p}\right)$ with average rates of 85%. However, with the same premise discussed above, $\left(N_{5}^{h},N_{5}^{p}\right)$ and $\left(N_{6}^{h},N_{6}^{p}\right)$ were better choices than $\left(N_{7}^{h},N_{7}^{p}\right)$. Using these two sets yielded more comparable performance between patient and healthy group. Finally, $\left(N_{6}^{h},N_{6}^{p}\right)$ was selected with *t* = 9 as the training set for the proposed model.

**Table 4:** Comparison of the best performances between different numbers of training subjects

***t***	**(*N***^***h***^,***N***^***p***^**)**	***r***_***h***_	***r***_***p***_	***r***
**1**	(100,100)	75%	95%	85%
**9**	(80,80)	80%	90%	85%
**9**	(60,60)	80%	90%	85%
**6**	(48,48)	75%	85%	80%
**9**	(32,32)	75%	90%	83%
**5**	(24,24)	80%	75%	78%
**3**	(12,12)	70%	80%	75%

### Model evaluation

The model validation stage was used to determine optimal parameters for the prediction model, resulting in a combination of EMG signals recorded after stimulation of sites 9 and 10 and 80 training subjects for each group (*t* = 9). The optimized model was tested with the test set **TE**. The test set contained 40 subjects (20 patients and 20 healthy volunteers). Following the scheme in Figure 3, the evaluation with the test set returned an average prediction *r* = 80% with *r*_*h*_ = *r*_*p*_ = 80%. This means that with 40 query subjects, 8 were misclassified, in which 4 healthy subjects were misclassified as patients and 4 patients were misclassified as healthy subjects. Since the test set was evenly distributed with respect to gender and age, misclassified subjects were grouped based on these two factors to see how they affected prediction results. The following values were computed:

$$\begin{array}{l} \upsilon_{G_{1}}=\frac{\#\mathrm{misclassified}\;\mathrm{males}}{\#\mathrm{misclassified}\;\mathrm{subjects}}\left(\%\right)\\ \upsilon_{G_{2}}=\frac{\#\mathrm{misclassified}\;\mathrm{females}}{\#\mathrm{misclassified}\;\mathrm{subjects}}\left(\%\right)\\ \upsilon_{A_{1}}=\frac{\#\mathrm{misclassified}\;\mathrm{subjects}<50y}{\#\mathrm{misclassified}\;\mathrm{subjects}}\left(\%\right)\\ \upsilon_{A_{2}}=\frac{\#\mathrm{misclassified}\;\mathrm{subjects}\geq 50y}{\#\mathrm{misclassified}\;\mathrm{subjects}}\left(\%\right) \end{array}$$

Results revealed differences among misclassified subject based on both factors. There were 7 female subjects misclassified $\left(\upsilon_{G_{2}}=87.5\%\right)$. The age factor also showed rather strong influence on the prediction result: $\upsilon_{A_{2}}=75\%$ meaning that 6 out of 8 misclassified subjects were from the elder age group (age > 50 years).

### Comparison with current methods

Critical values to assess widespread central hyperexcitability using the NWR and RRF have recently been published [10]. In particular, estimates of 95^th^, 90^th^ and 75^th^ percentile values of the distribution of the test responses have been obtained (named p^95^, p^90^ and p^75^, respectively) by computing quantile regressions for the each assessment method (e.g. NWR thresholds, RRF areas). More extreme values (e.g. p^95^) are more likely to lead to the correct identification of patients, but could leave out a number of subjects that could also potentially be at risk, whereas critical values that correspond to more central percentiles of the distribution (e.g. p^75^) would include more subjects potentially at risk, but at the cost of misclassifying more healthy subjects as presenting hyperexcitability.

With the present dataset, it was possible to compute individual RRF areas for chronic pain patients and healthy subjects (for details, please refer to [27]), and compare them to these critical values to obtain equivalent classification rates *r*_*p*_, *r*_*h*_ and *r* using the same test set **TE**. The classification rates for the most restrictive conditions (p^95^ and p^90^) were *r*_*p*_ = 0%, *r*_*h*_ = 100% and *r* = 50%, whereas for the least restrictive condition (p^75^) the classification rates were *r*_*p*_ = 20%, *r*_*h*_ = 95% and *r* = 57.5%. This means that the distribution of RRF areas of patients and healthy controls largely overlap resulting in criteria that is too restrictive for detecting central hyperexcitability, as evidenced by the very low classification rates for patients obtained with this method.

## Discussion

Experimental and clinical studies in diverse cohorts of patients (e.g., whiplash, fibromyalgia, osteoarthritis, musculoskeletal disorders, headache, and neuropathic, visceral and post-surgical pain) have shown that these pathologies share common features, which are likely to reflect alterations in central nociceptive processing [28,29] leading to exaggerated pain sensitivity. It has been previously established that changes in central nociceptive processing can be detected by electrophysiological tests, such as those based on the NWR. In the past, the NWR has been widely used as a biomarker in the assessment of the state of the nociceptive system [5,6,30], and it has been proposed as a key tool in the research of central sensitization mechanisms, which are believed to be linked to the development of chronic pain [5,7,8,31,32].

In this regard, a number of studies showed that several patient groups present lower NWR thresholds compared to control groups of healthy volunteers [7,8,33,34]. Moreover, it was also demonstrated that chronic pain patients (endometriosis, chronic low back and chronic neck pain) present larger RRF compared to pain free subjects [12,31]. Lower NWR thresholds and enlargement of RRF are objective signs of central hyperexcitability, which could be a consequence of increased number of responsive spinal neurons or an expansion of the receptive fields of spinal neurons as a result of increased synaptic sensitivity [35,36]. In the light of these facts, there is clear evidence that groups of patients with different chronic pain conditions display on average altered central pain processing. However, the next translational step in this field involves the definition of diagnostic criteria in individual patients, in order to develop treatments that are tailored to detect individual disturbances in central pain processing [29].

In this study, a set of features derived from the NWR of chronic low back and neck pain patients and healthy volunteers was used as input to a prediction model, in order to test the hypothesis that the NWR contains specific information that would allow individual classification regarding the condition of the test subjects. Several features derived from the NWR have been used in the past for detection or quantification purposes: NWR latencies, raw EMG amplitudes, mean and peak EMG values, EMG probability distribution, EMG root-mean-square (RMS), z-scores and RRF area size, among others [6,27,32,37-41]. Additionally, other EMG features have also been used in classifications tasks in other fields (most notably myoelectric control systems), such as number of zeros crossings, slope sign changes, spectral moments, as well as frequency domain and time-scale features [42-44].

A preliminary analysis showed that, among these variables, EMG probability distributions showed the most promising results in terms of discriminating potential for classifying between patients and healthy volunteers. Thus, they were selected for further development of the prediction model. However, the EMG signals showed a rather large range, thus requiring a high number of bins for their histogram representation. In order to overcome this, a new range was defined, restricting the original range around one standard deviation of the mean, In this way, less bins were required for the representation (as a simple method of feature selection), effectively reducing the number of features to be fed to the prediction model.

The evaluation of stimulation sites for eliciting the NWR revealed that EMG signals recorded after stimulation of electrodes located in the anterolateral (site 3) and heel (sites 9 and 10) regions and of the sole of the foot presented the best prediction rates. This is in accordance with previous research showing that the RRF in chronic low back and neck pain patients are expanded compared to healthy volunteers, precisely towards these regions [31,45]. On the other hand, EMG signals recorded after stimulation of sites located at or around the arch on the sole of the foot resulted in the worst prediction rates. These locations often have thin skin layer which lead to higher pain sensitivity and large reflexes regardless of whether they are patients or healthy subjects [46].

The final model evaluation showed an average prediction rate of 80%. For that particular choice of model, there were no differences in the misclassification rates between healthy subjects and patients. A more detailed analysis of the results focusing on the demographics of the two groups, revealed that women and elder subjects are more likely to be misclassified using the selected model. To date, there are no studies describing age or gender differences in EMG signals recorded from chronic pain patients compared healthy volunteers in relation to the NWR, since most of the research is focused on other biomarkers, most notably the NWR thresholds to single and repeated stimulation (temporal summation), and the RRF areas [9,26,32]. In this regard, there is still no agreement on the effects of age and gender on the NWR, although most of the evidence seems to point towards generally lower NWR thresholds in women and elderly subjects, most likely due to reduced endogenous analgesic mechanisms [6,10,47,48].

To date, only population differences have been reported between chronic low back and neck pain patients compared to healthy volunteers, showing an enlargement of the RRF in patients [12]. More recently, however, reference ranges for the NWR and RRF have been established for healthy subjects [10]. These ranges establish critical values for several parameters derived from the NWR (e.g. NWR threshold to single and repeated electrical stimulation, RRF area), above which an individual subject can be considered to present widespread central hyperexcitability. Results using this method with RRF areas as the classification parameters showed lower average classification rates (*r* = 57.5%) and very low classification rates for patients (*r*_*p*_ = 20%) compared to the prediction model. This is most likely due to the large inter-individual variation of the RRF areas and the high overlap that exists between the probability distribution of RRF areas in patients and healthy subjects.

### Limitations and future work

This work focused on the assessment of central hyperexcitability in individual chronic low back and neck pain patients using the NWR. Although the quantification of the NWR does not rely on subjective self-reports of pain sensation, it is subjected to supraspinal modulation. External factors involving affective and cognitive processes or other ongoing nociceptive processes (e.g. endogenous pain modulatory mechanisms) can affect the NWR characteristics [6,24], so these factors have to be carefully controlled for in order to provide reliable outcomes. Further tests in other patient groups should be conducted in order to test if this model could also be used to characterize other pain conditions.

Furthermore, this is the first known attempt at individualized classification between healthy subjects and chronic pain patients based on the assessment of central hypersensitivity provided by the NWR. As it is common in classification tasks, there are several variables that require a careful selection, such as the choice of features to be used as input to the prediction model (in this case, the EMG probability distribution), the parameters of the classifier (for kNN, the number of neighbours), the size of the datasets for classification, validation and test, and the number and location of stimulation sites selected. Some of these variables were chosen based on prior knowledge and/or empirical tests, so whereas the proposed statistical model is able to achieve high prediction rates, future research could focus on the application of more advanced signal processing methods, e.g. alternative methods for feature generation and selection, adaptive histograms, adaptive kernel density estimators and optimal parameter selection for the classifier, among others.

## Conclusions

A prediction model was proposed as a new approach for objective and individual assessment of central hyperexcitability in the nociceptive system. The model was developed using statistical properties of EMG signals recorded after eliciting the nociceptive withdrawal reflex. The model supports individualized assessment of patients, including an estimation of the confidence of the predicted result. Evaluation was carried out using an independent test set of healthy subjects and chronic pain patients and a high prediction rate of 80% was achieved. Therefore, the present statistical prediction model constitutes a first step towards potential applications in clinical practice.

## Competing interests

The authors declare that they have no competing interests.

## Authors’ contributions

JBM, MC, TBM and OKA defined the research topic. MC provided the datasets used in the analysis. GPN developed the presented methodology. GPN and JBM analyzed the data, interpreted the results and drafted the manuscript. MC, TBM and OKA contributed to the analysis, interpretation, and presentation of the manuscript. All authors have read and approved the final version of the manuscript.

## References

[B1] Woolf CJ Salter MW Neuronal Plasticity: Increasing the Gain in Pain Science 2000 288 5472 1765 1768 10.1126/science.288.5472.1765 10846153

[B2] Curatolo M Arendt-Nielsen L Petersen-Felix S Evidence, mechanisms, and clinical implications of central hypersensitivity in chronic pain after whiplash injury Clin J Pain 2004 20 6 469 476 10.1097/00002508-200411000-00013 15502692

[B3] Borsook D Neurological diseases and pain Brain 2012 135 2 320 344 10.1093/brain/awr271 22067541 PMC3281476

[B4] U.S. Department Of Health And Human Services National Institutes Of Health, American Recovery And Reinvestment Act Of 2009, Challenge Grant Applicationshttp://grants.nih.gov/grants/funding/challenge_award/omnibus.pdf

[B5] Skljarevski V Ramadan NM The nociceptive flexion reflex in humans - review article Pain 2002 96 1–2 3 8 11932055 10.1016/s0304-3959(02)00018-0

[B6] Sandrini G Serrao M Rossi P Romaniello A Cruccu G Willer JC The lower limb flexion reflex in humans Prog Neurobiol 2005 77 6 353 395 10.1016/j.pneurobio.2005.11.003 16386347

[B7] Banic B Petersen-Felix S Andersen OK Radanov BP Villiger PM Arendt-Nielsen L Curatolo M Evidence for spinal cord hypersensitivity in chronic pain after whiplash injury and in fibromyalgia Pain 2004 107 1–2 7 15 14715383 10.1016/j.pain.2003.05.001

[B8] Desmeules JA Cedraschi C Rapiti E Baumgartner E Finckh A Cohen P Dayer P Vischer TL Neurophysiologic evidence for a central sensitization in patients with fibromyalgia Arthritis Rheum 2003 48 5 1420 1429 10.1002/art.10893 12746916

[B9] Neziri AY Andersen OK Petersen-Felix S Radanov B Dickenson AH Scaramozzino P Arendt-Nielsen L Curatolo M The nociceptive withdrawal reflex: Normative values of thresholds and reflex receptive fields Eur J Pain 2010 14 2 134 141 10.1016/j.ejpain.2009.04.010 19505833

[B10] Scaramozzino P Neziri AY Andersen OK Arendt-Nielsen L Curatolo M Percentile normative values of parameters of electrical pain and reflex thresholds Scand J Pain 2013 4 2 120 124 10.1016/j.sjpain.2012.09.002 29913899

[B11] Neziri AY Dickenmann M Scaramozzino P Andersen OK Arendt-Nielsen L Dickenson AH Curatolo M Effect of intravenous tropisetron on modulation of pain and central hypersensitivity in chronic low back pain patients Pain 2012 153 2 311 318 10.1016/j.pain.2011.10.008 22100357

[B12] Biurrun Manresa JA Neziri AY Curatolo M Arendt-Nielsen L Andersen OK Reflex receptive fields are enlarged in patients with musculoskeletal low back and neck pain Pain 2013 in press, doi:10.1016/j.pain.2013.04.013 10.1016/j.pain.2013.04.013 23707309

[B13] Duda R Hart P Stork D Pattern Classification and Scene Analysis 2000 2 New York: John Wiley & Sons

[B14] Biurrun Manresa JA Hansen J Andersen OK Development of a data acquisition and analysis system for nociceptive withdrawal reflex and reflex receptive fields in humans Conf Proc IEEE Eng Med Biol Soc 2010 2010 6619 6624 21096727 10.1109/IEMBS.2010.5627139

[B15] Phyu TN Ao SI, Castillo O, Douglas C, Feng DD, Lee JA Survey of Classification Techniques in Data Mining Proceedings of the International MultiConference of Engineers and Computer Scientists 2009 2009 I Hong Kong: Newswood Limited 727 731

[B16] Arlot S Celisse A A survey of cross-validation procedures for model selection Stat Surv 2010 4 40 79 10.1214/09-SS054

[B17] Hagberg M The amplitude distribution of surface EMG in static and intermittent static muscular performance Eur J Appl Physiol Occup Physiol 1979 40 4 265 272 10.1007/BF00421518 428378

[B18] Harba MIA Ibraheem AA EMG processor based on the amplitude probability distribution J Biomed Eng 1986 8 2 105 114 10.1016/0141-5425(86)90044-0 3713141

[B19] Linderhed H A new dimension to amplitude analysis of EMG Int J Ind Ergon 1993 11 3 243 247 10.1016/0169-8141(93)90112-Q

[B20] Reaz MBI Hussain MS Mohd-Yasin F Techniques of EMG signal analysis: Detection, processing, classification and applications Biol Proc Online 2006 8 1 11 35 10.1251/bpo115 PMC1455479 16799694

[B21] Cover T Hart P Nearest neighbor pattern classification Information Theory, IEEE Transactions on 1967 13 1 21 27

[B22] Zhang P Model Selection Via Multifold Cross Validation The Annals of Statistics 1993 21 1 299 313 10.1214/aos/1176349027

[B23] Kulkarni SR Lugosi G Venkatesh SS Learning pattern classification-A survey IEEE Trans Inf Theory 1998 44 6 2178 2206 10.1109/18.720536

[B24] Andersen OK Studies of the organization of the human nociceptive withdrawal reflex. Focus on sensory convergence and stimulation site dependency Acta Physiol 2007 189 1 35 10.1111/j.1748-1716.2007.01706.x 17439638

[B25] Kotsiantis SB Supervised machine learning: A review of classification techniques Inf 2007 31 3 249 268

[B26] Neziri AY Curatolo M Nüesch E Scaramozzino P Andersen OK Arendt-Nielsen L Jüni P Factor analysis of responses to thermal, electrical, and mechanical painful stimuli supports the importance of multi-modal pain assessment Pain 2011 152 5 1146 1155 10.1016/j.pain.2011.01.047 21396782

[B27] Neziri AY Curatolo M Bergadano A Petersen-Felix S Dickenson A Arendt-Nielsen L Andersen OK New method for quantification and statistical analysis of nociceptive reflex receptive fields in humans J Neurosci Methods 2009 178 1 24 30 10.1016/j.jneumeth.2008.11.009 19063920

[B28] Woolf CJ Central sensitization: Implications for the diagnosis and treatment of pain Pain 2011 152 SUPPL.3 S2 S15 20961685 10.1016/j.pain.2010.09.030 PMC3268359

[B29] Curatolo M Diagnosis of altered central pain processing Spine 2011 36 25 Suppl S200 204 22020613 10.1097/BRS.0b013e3182387f3d

[B30] Peters ML Schmidt AJM Van Den Hout MA Koopmans R Sluijter ME Chronic back pain, acute postoperative pain and the activation of diffuse noxious inhibitory controls (DNIC) Pain 1992 50 2 177 187 10.1016/0304-3959(92)90159-9 1408314

[B31] Neziri AY Haesler S Petersen-Felix S Müller M Arendt-Nielsen L Manresa JB Andersen OK Curatolo M Generalized expansion of nociceptive reflex receptive fields in chronic pain patients Pain 2010 151 3 798 805 10.1016/j.pain.2010.09.017 20926191

[B32] Neziri AY Curatolo M Limacher A Nüesch E Radanov B Andersen OK Arendt-Nielsen L Jüni P Ranking of parameters of pain hypersensitivity according to their discriminative ability in chronic low back pain Pain 2012 153 10 2083 2091 10.1016/j.pain.2012.06.025 22846347

[B33] Perrotta A Sandrini G Serrao M Buscone S Tassorelli C Tinazzi M Zangaglia R Pacchetti C Bartolo M Pierelli F Martignoni E Facilitated temporal summation of pain at spinal level in Parkinson's disease Mov Disord 2011 26 3 442 448 10.1002/mds.23458 21462260

[B34] Sterling M Differential development of sensory hypersensitivity and a measure of spinal cord hyperexcitability following whiplash injury Pain 2010 150 3 501 506 10.1016/j.pain.2010.06.003 20594646

[B35] Cook AJ Woolf CJ Wall PD McMahon SB Dynamic receptive field plasticity in rat spinal cord dorsal horn following C-primary afferent input Nature 1987 325 6100 151 153 10.1038/325151a0 3808072

[B36] Dubner R Basbaum AI Wall PD, Melzack R Spinal dorsal horn plasticity following tissue or nerve injury Textbook of Pain 1994 3 Edinburgh: Churchill Livingstone 225 241

[B37] Andersen OK Spaich EG Madeleine P Arendt-Nielsen L Gradual enlargement of human withdrawal reflex receptive fields following repetitive painful stimulation Brain Res 2005 1042 2 194 204 10.1016/j.brainres.2005.02.039 15854591

[B38] Arendt-Nielsen L Sonnenborg FA Andersen OK Facilitation of the withdrawal reflex by repeated transcutaneous electrical stimulation: An experimental study on central integration in humans Eur J Appl Physiol Occup Physiol 2000 81 3 165 173 10.1007/s004210050026 10638373

[B39] Biurrun Manresa JA Jensen MB Andersen OK Introducing the reflex probability maps in the quantification of nociceptive withdrawal reflex receptive fields in humans J Electromyogr Kinesiology 2011 21 1 67 76 10.1016/j.jelekin.2010.09.003 20934351

[B40] France CR Rhudy JL McGlone S Using normalized EMG to define the nociceptive flexion reflex (NFR) threshold: Further evaluation of standardized NFR scoring criteria Pain 2009 145 1–2 211 218 19595510 10.1016/j.pain.2009.06.022

[B41] Rhudy JL France CR Defining the nociceptive flexion reflex (NFR) threshold in human participants: A comparison of different scoring criteria Pain 2007 128 3 244 253 10.1016/j.pain.2006.09.024 17070999 PMC1993909

[B42] Asghari Oskoei M Hu H Myoelectric control systems-A survey Biomed Signal Process Control 2007 2 4 275 294 10.1016/j.bspc.2007.07.009

[B43] Hudgins B Parker P Scott RN A new strategy for multifunction myoelectric control IEEE Trans Biomed Eng 1993 40 1 82 94 10.1109/10.204774 8468080

[B44] Parker P Englehart K Hudgins B Myoelectric signal processing for control of powered limb prostheses J Electromyogr Kinesiology 2006 16 6 541 548 10.1016/j.jelekin.2006.08.006 17045489

[B45] Neziri AY Manresa JB Jüni P Arendt-Nielsen L Andersen OK Curatolo M Expansion of nociceptive reflex receptive fields in patients with low back and neck pain Eur J Pain Suppl 2011 5 1 109 110

[B46] Jain AK Duin RPW Mao J Statistical pattern recognition: A review IEEE Trans Pattern Anal Mach Intell 2000 22 1 4 37 10.1109/34.824819

[B47] Serrao M Rossi P Sandrini G Parisi L Amabile GA Nappi G Pierelli F Effects of diffuse noxious inhibitory controls on temporal summation of the RIII reflex in humans Pain 2004 112 3 353 360 10.1016/j.pain.2004.09.018 15561391

[B48] Edwards RR Fillingim RB Ness TJ Age-related differences in endogenous pain modulation: A comparison of diffuse noxious inhibitory controls in healthy older and younger adults Pain 2003 101 1–2 155 165 12507710 10.1016/s0304-3959(02)00324-x

